# Identification of a competing endogenous RNA network related to immune signature in clear cell renal cell carcinoma

**DOI:** 10.18632/aging.203784

**Published:** 2021-12-27

**Authors:** Yuke Zhang, Jiangwen Dai, Weifeng Huang, Qingsong Chen, Wei Chen, Qiying He, Feng Chen, Peng Zhang

**Affiliations:** 1 Department of Urology, West China Hospital of Sichuan University, Chengdu, China; 2 Department of Oncology, Chengdu Fifth People’s Hospital of Chengdu University of TCM, Chengdu, China; 3 Department of Hepatobiliary Surgery, The First Affiliated Hospital of Chongqing Medical University, Chongqing, China; 4 Department of Traumatology, Chongqing University Central Hospital, Chongqing, China; 5 Department of Hepatobiliary Surgery, Daping Hospital, Army Medical University, Chongqing, China; 6 Department of Integrated Care Management Center, West China Hospital of Sichuan University, Chengdu, China

**Keywords:** clear cell renal cell carcinoma, competing endogenous RNA, tumor microenvironment, prognosis, immune cell infiltration

## Abstract

Clear cell renal cell carcinoma (ccRCC) is a fatal cancer of the urinary system. Long non-coding RNAs (lncRNAs) act as competitive endogenous RNAs (ceRNAs) involving the ccRCC progression. However, the relationship between the ceRNA network and immune signature is largely unknown. In this study, the ccRCC-related gene expression profiles retrieved from the TCGA database were used first to identify the differentially expressed genes through differential gene expression analysis and weighted gene co-expression network analysis. The interaction among differentially expressed lncRNAs, miRNAs, and mRNAs were matched using public databases. As a result, a ceRNA network was developed that contained 144 lncRNAs, 23 miRNAs, as well as 62 mRNAs. Four of 144 lncRNAs including LINC00943, SRD5A3-AS1, LINC02345, and U62317.3 were identified through LASSO regression and Cox regression analyses, and were used to create a prognostic risk model. Then, the ccRCC samples were divided into the high- and low-risk groups depending on their risk scores. ROC curves, Kaplan-Meier survival analysis, and the survival risk plots indicated that the predictive performance of our developed risk model was accurate. Moreover, the CIBERSORT algorithm was used to measure the infiltration levels of immune cells in the ccRCC samples. The further genomic analysis illustrated a positive correlation between most immune checkpoint blockade-related genes and the risk score. In conclusion, the present findings effectually contribute to the comprehensive understanding of the ccRCC pathogenesis, and may offer a reference for developing novel therapeutic and prognostic biomarkers.

## INTRODUCTION

Renal cell carcinoma (RCC) has been known to be among the most fatal cancers in the urinary system [1]. RCC consists of three major histological subtypes, including chromophobe RCC, papillary RCC, and clear cell renal cell carcinoma (ccRCC). ccRCC is reported to be responsible for ~ 80% of total RCC incidence [2, 3]. Surgical resection is the principal treatment strategy, as ccRCC patients are insensitive to chemotherapy and radiotherapy. However, a third of the patients who undergo surgery have a recurrence, further leading to poor prognosis and high mortality [4]. Although in recent years, several targeted therapeutics such as sorafenib, sunitinib, and everolimus have successfully obtained approval for the clinical application in ccRCC treatment, their treatment effects are variable and the patients’ conditions usually deteriorate, which necessitates further investigation for identifying new therapeutic agents as well as prognostic biomarkers [5, 6].

Long non-coding RNAs (lncRNAs) are non-coding RNAs (ncRNAs) with a length of >200 nucleotides, and exert fundamental contributions to a variety of biological processes, such as chromatin organization, transcriptional regulation, and posttranscriptional modification [7]. The highly conserved small ncRNAs known as microRNAs (miRNAs) have the length of 20-25 nucleotides [8]. The competitive endogenous RNA (ceRNA) concept describes the function of lncRNAs, messenger RNAs (mRNAs), and other RNA transcript types as natural miRNA “sponges” that suppress miRNA functions via miRNA response elements (MREs) [9]. lncRNAs can reduce the affinity between miRNAs and mRNAs by competing for miRNA binding, causing aberrant expressions of mRNAs. Several studies have validated the ceRNA hypothesis, and it is widely recognized as tightly associated with the initiation and progression of various cancers [10, 11].

As an intricate and complex ecosystem, the tumor microenvironment (TME) comprises a variety of innate and adaptive immune cells along with the cancer cells and the surrounding stroma [12, 13]. The infiltration of immune cells contributes to various aspects of tumor progression and is closely related to clinical outcomes [14]. For instance, the infiltration of CD8+ T cells indicates a better prognosis and response to immunotherapy in many cancers [15]. However, persistent antigen exposure reduces the propagation and cytotoxicity of CD8+ T cells, leading to an ‘exhaustion’ phenotype. The exhausted state is accompanied by the enhanced expression levels of immune checkpoint blockade (ICB)-related genes, including PD-1, TIM3, and CTLA4. Blockade of these molecules can rescue the exhaustion phenotype [16]. Macrophages are a major component of immune cell types in TME. They can be activated and polarized into two diverse subtypes, the M1 and M2 macrophages, through differential stimulation by cytokines and chemokines [17]. M1 macrophages are characterized by their ability to release abundant proinflammatory cytokines, efficient antigen presentation, and tumoricidal activity [18]. On the contrary, M2 macrophages inhibit inflammatory responses and create an immunosuppressive microenvironment for tumor growth, metastasis, and angiogenesis [19]. Taken together, a better understanding of infiltration of the immune cells is a substantial step toward the diagnosis and treatment of ccRCC.

In the present study, the gene expression profiles were retrieved from The Cancer Genome Atlas (TCGA) database. The obtained data were then evaluated by differential gene expression analysis as well as weighted gene co-expression network analysis (WGCNA). Then, public databases were applied for examining the interactions among lncRNAs, miRNAs, and mRNAs, and constructing a ceRNA network. The genes within the ceRNA network were then analyzed by gene ontology (GO) and Kyoto Encyclopedia of Genes and Genomes (KEGG) pathway analyses, and the potential pathogenesis mechanisms of ccRCC were identified. Consequently, four lncRNAs were utilized to create a prognostic risk model with a validated good predictive accuracy. The ccRCC samples were graded into the high- and low-risk groups. The high-risk group-related genes were identified, and underwent functional enrichment analysis. Finally, the infiltration levels of immune cells and the expression levels of ICB-related genes were measured, and compared in different risk groups. In summary, our study is envisaged to produce novel insights about the carcinogenesis mechanisms of ccRCC, and provide a reliable reference for developing therapeutic targets for it.

## RESULTS

### Differential gene expression analysis

**Figure 1:**
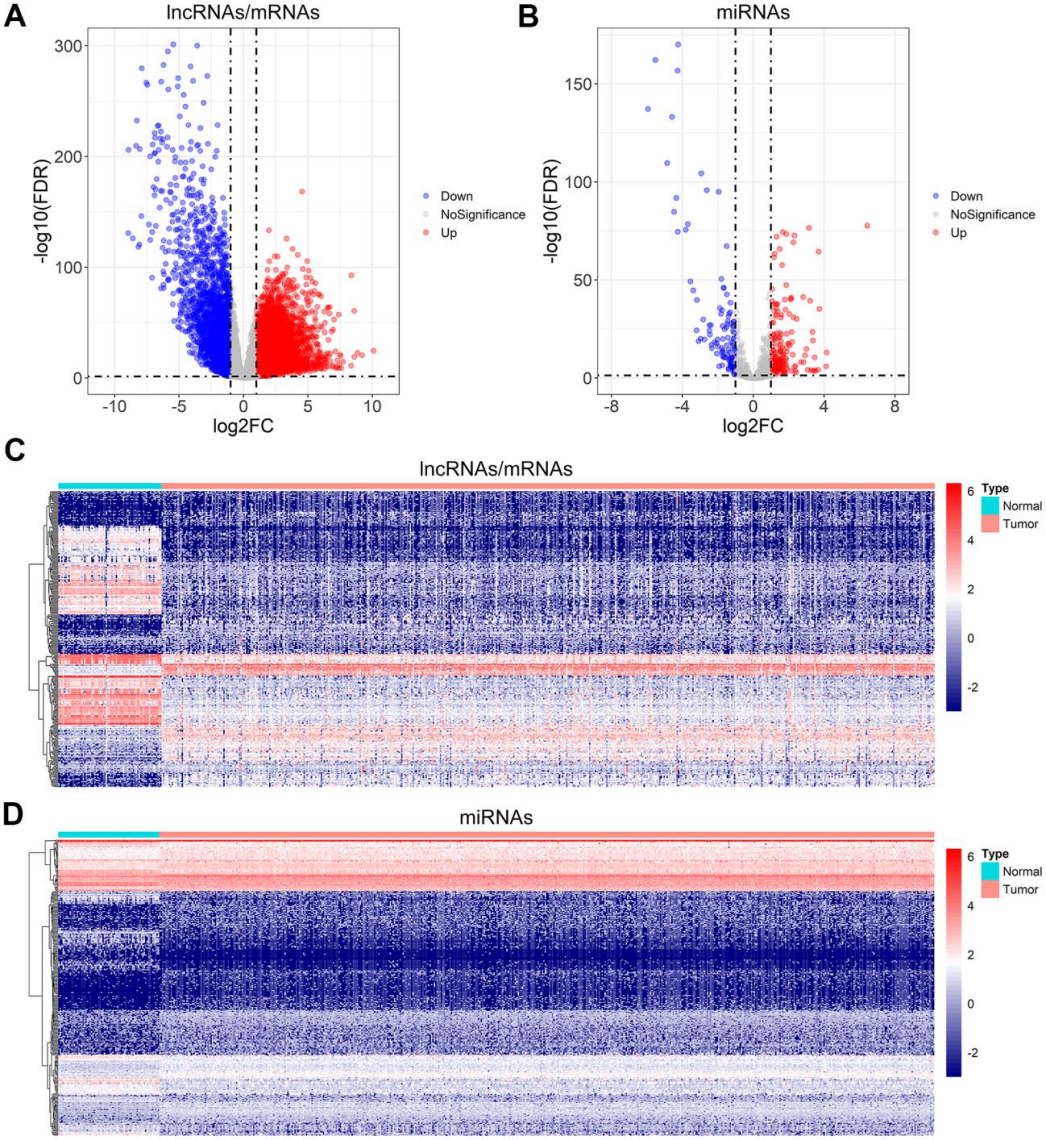
**Differential gene expression analysis for ccRCC.** (**A**, **B**) Volcano plot of the differentially expressed lncRNAs/mRNAs, and miRNAs. (**C**, **D**) The heatmap of the top 300 differentially expressed lncRNAs/mRNAs, and miRNAs.

In order to detect the differentially expressed lncRNAs, miRNAs, and mRNAs, the TCGA database was searched for the RNA sequencing data of ccRCC samples. A total of 2653 differentially expressed lncRNAs (2040 up-regulated and 613 down-regulated), 316 differentially expressed miRNAs (202 up-regulated and 114 down-regulated), and 4971 differentially expressed mRNAs (3055 up-regulated and 1916 down-regulated) were identified using the set threshold of |log2FC (fold change)| > 1 and false discovery rate (FDR) < 0.05. The Volcano plots and heatmaps were performed to visualize and compare the distribution of the differentially expressed lncRNAs/mRNAs (Figure 1A, 1C) and differentially expressed miRNAs (Figure 1B, 1D) between ccRCC and normal renal samples.

### Construction of the weighted gene co-expression network for lncRNAs and mRNAs

**Figure 2:**
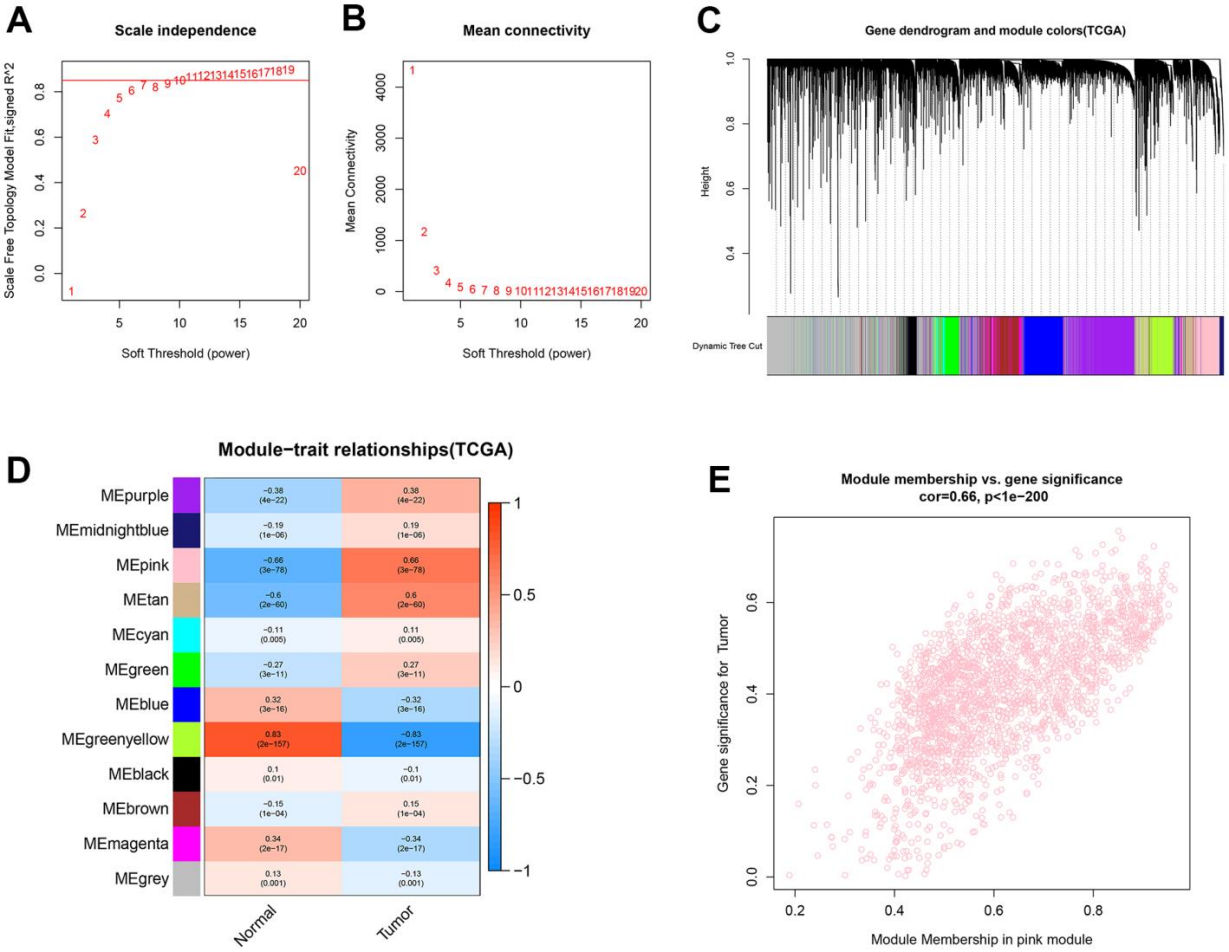
**Identification of co-expression modules for lncRNAs/mRNAs based on WGCNA.** (**A**, **B**) The scale-free fit index and the mean connectivity for various soft-thresholding powers (β) were optimized. (**C**) Cluster dendrogram of lncRNAs/mRNAs based on the 1-TOM. (**D**) Heatmap of the correlation between module eigengenes and sample types. (**E**) Scatter plot of module eigengenes for ccRCC samples in the pink module.

Functional gene clusters in ccRCC samples were determined using the WGCNA package. With a soft-threshold β set at 11, a scale-free network was obtained with the scale-free R^2^ = 0.86 (Figure 2A, 2B). Then, 12 modules were detected after merging the highly similar co-expression modules (Figure 2C). Among them, the highest positive correlation with ccRCC samples belonged to the pink module, with the module membership (MM) and gene significance (GS) correlation of 0.66 (Figure 2D, 2E). Finally, 359 lncRNAs and 1499 mRNAs were extracted from the pink module for further analysis.

### Construction of the weighted gene co-expression network for miRNAs

**Figure 3:**
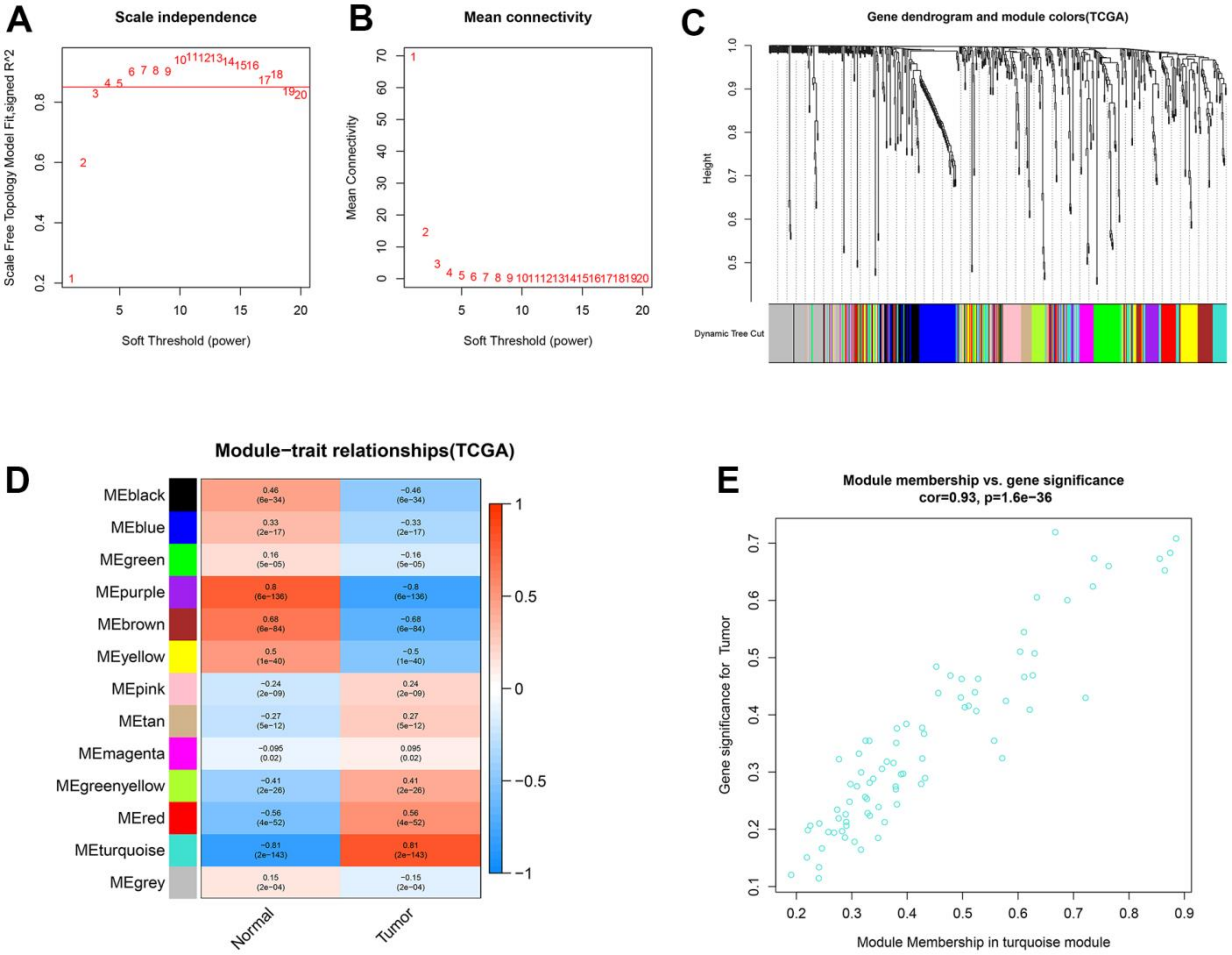
**Identification of co-expression modules for miRNAs by WGCNA.** (**A**, **B**) The scale-free fit index and the mean connectivity for various soft-thresholding powers (β) were optimized. (**C**) Cluster dendrogram of miRNAs based on the 1-TOM. (**D**) Heatmap of the correlation between module eigengenes and sample types. (**E**) Scatter plot of module eigengenes for ccRCC samples in the turquoise module.

The weighted co-expression network was developed for the miRNAs using the same method as described for lncRNAs and mRNAs. The β values were optimized, and β = 4 (R^2^ = 0.86) was chosen as the optimum (Figure 3A, 3B). A total of 611 miRNAs were clustered into 13 modules (Figure 3C). Among these identified modules, the turquoise module, containing 83 miRNAs, was substantially related to ccRCC samples (Figure 3D). Additionally, a high GS-MM correlation (0.93) was identified in the turquoise module (Figure 3E).

### Construction of the ceRNA network

**Figure 4:**
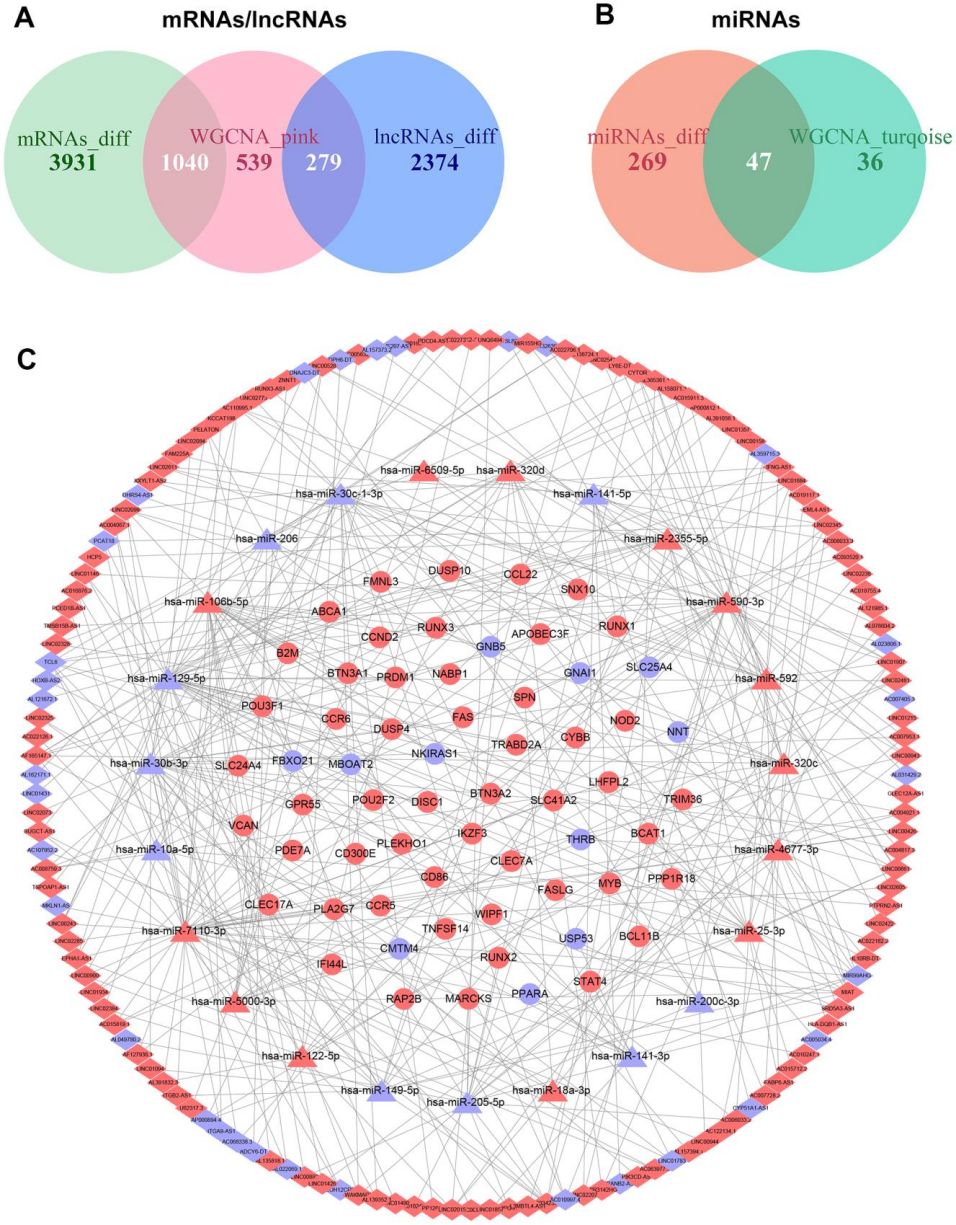
**Construction of the ceRNA network in ccRCC.** (**A**) Overlapping mRNAs and lncRNAs between differential gene expression analysis and WGCNA. (**B**) Overlapping miRNAs between differential gene expression analysis and WGCNA. (**C**) The ceRNA network was comprised of 144 lncRNAs, 23 miRNAs, and 62 mRNAs; red nodes represent up-regulation, and blue nodes represent down-regulation; diamond nodes represent lncRNAs, triangle nodes represent miRNAs, and ellipse nodes represent mRNAs.

We constructed the ceRNA network to investigate the regulatory mechanisms underlying lncRNAs in ccRCC. First, a total of 279 overlapping lncRNAs, 47 overlapping miRNAs, and 1040 overlapping mRNAs were identified through the intersection of differential gene expression analysis and WGCNA (Figure 4A, 4B). Then, the interactions between 1040 lncRNAs and 279 miRNAs were investigated using LncBase v.2 database. As a result, 457 interacting pairs of lncRNAs and miRNAs were identified, consisting of 173 lncRNAs and 45 miRNAs. Next, the three databases of TargetScan, miRDB, and miRTarBase were applied to assess mRNAs targeted by the 279 identified miRNAs in the previous stage. 73 interacting pairs of miRNAs and mRNAs were obtained, consisting of 23 miRNAs and 62 mRNAs. Finally, the above findings were applied to construct a lncRNAs-based ceRNA network. The ceRNA network is comprised of 144 lncRNAs, 23 miRNAs, and 62 mRNAs (Figure 4C).

### Functional enrichment analysis for mRNAs in the ceRNA network

**Figure 5:**
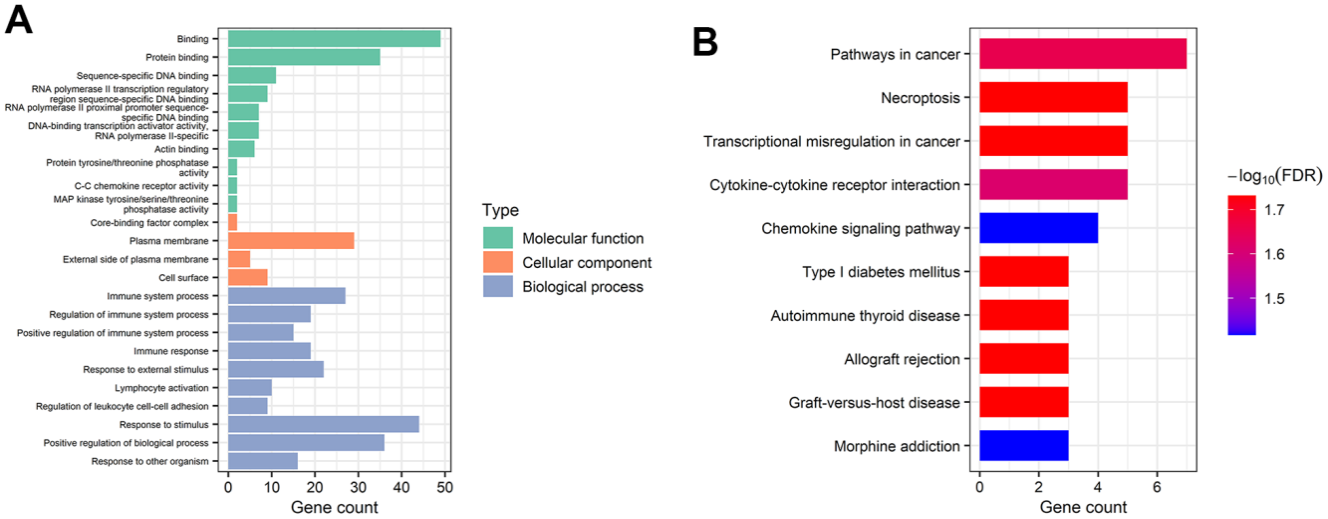
**Gene functional enrichment analysis of mRNAs in the ceRNA network.** (**A**) Overrepresented GO terms for MF, CC, and BP. (**B**) The top 10 functionally enriched pathways were identified by KEGG analysis.

GO and KEGG analyses were applied by utilizing STRING database for better comprehension of the potential biological functions and roles of the extracted mRNAs in the ceRNA network. In this regard, the terms “binding”, “protein binding”, “sequence-specific DNA binding”, and “RNA polymerase II transcription regulatory region sequence-specific DNA binding” were identified as significant under molecular function (MF). In addition, the mRNAs were mainly enriched in “core-binding factor complex”, “plasma membrane”, “external side of plasma membrane”, and “cell surface” in terms of cellular component (CC). Finally, the mRNAs related to biological process (BP) were most relevant in immune-related pathways, including “immune system process”, “regulation of immune system process”, “positive regulation of immune system process”, and “immune response” terms (Figure 5A). According to the KEGG pathway analysis, mRNAs became significantly enriched in “pathways in cancer”, “necroptosis”, “transcriptional misregulation in cancer”, “cytokine-cytokine receptor interaction”, and “chemokine signaling pathway” (Figure 5B).

### Development of a prognostic risk model by lncRNAs in the ceRNA network

**Figure 6:**
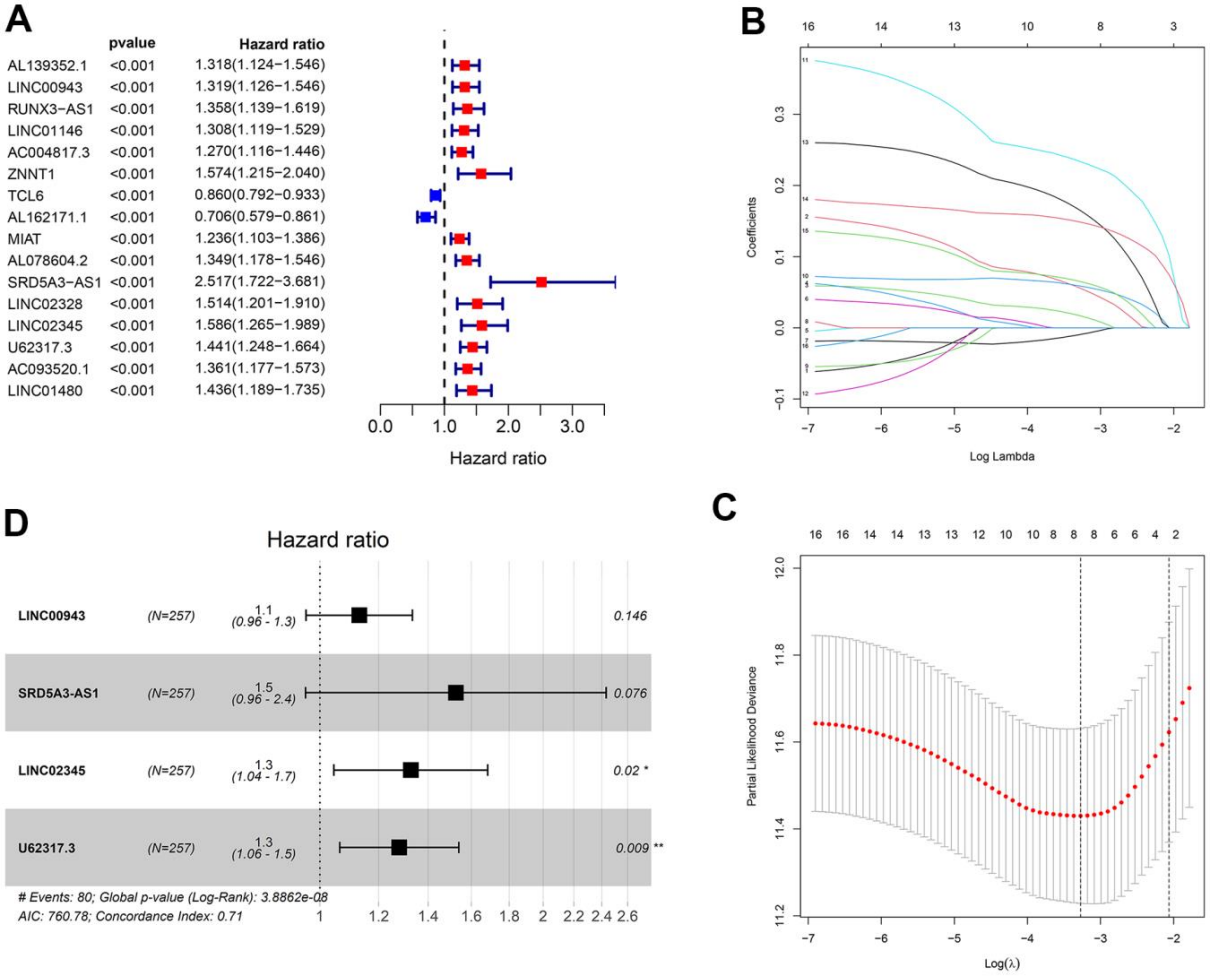
**Construction of the prognostic risk model.** (**A**) The results of the univariate Cox regression analysis of lncRNAs with p < 0.001. (**B**, **C**) Lambda.min value = 8 was calculated using LASSO regression. (**D**) The results of multivariable Cox regression analysis.

At first, ccRCC samples were classified in random groups for the training (n = 257) and the testing (n = 256) cohorts. Then, we developed the prognostic risk model using the training cohort. A total of 144 lncRNAs in the ceRNA network were evaluated via the univariate Cox regression analysis. With the threshold of p < 0.001, 16 lncRNAs were selected for further least absolute shrinkage and selection operator (LASSO) regression analysis. Eight lncRNAs were identified using lambda.min value (Figure 6A–6C), and then were integrated into the multivariate Cox regression analysis. Four lncRNAs with strong prognostic values were identified in total: LINC00943, SRD5A3-AS1, LINC02345, and U62317.3 (Figure 6D). Using these four lncRNAs, a prognostic risk model was developed with the calculated risk score = (0.122 * LINC00943 expression level) + (0.422 * SRD5A3-AS1 expression level) + (0.282 * LINC02345 expression level) + (0.247 * U62317.3 expression level).

### Evaluation of the prognostic signature in the training cohort

**Figure 7:**
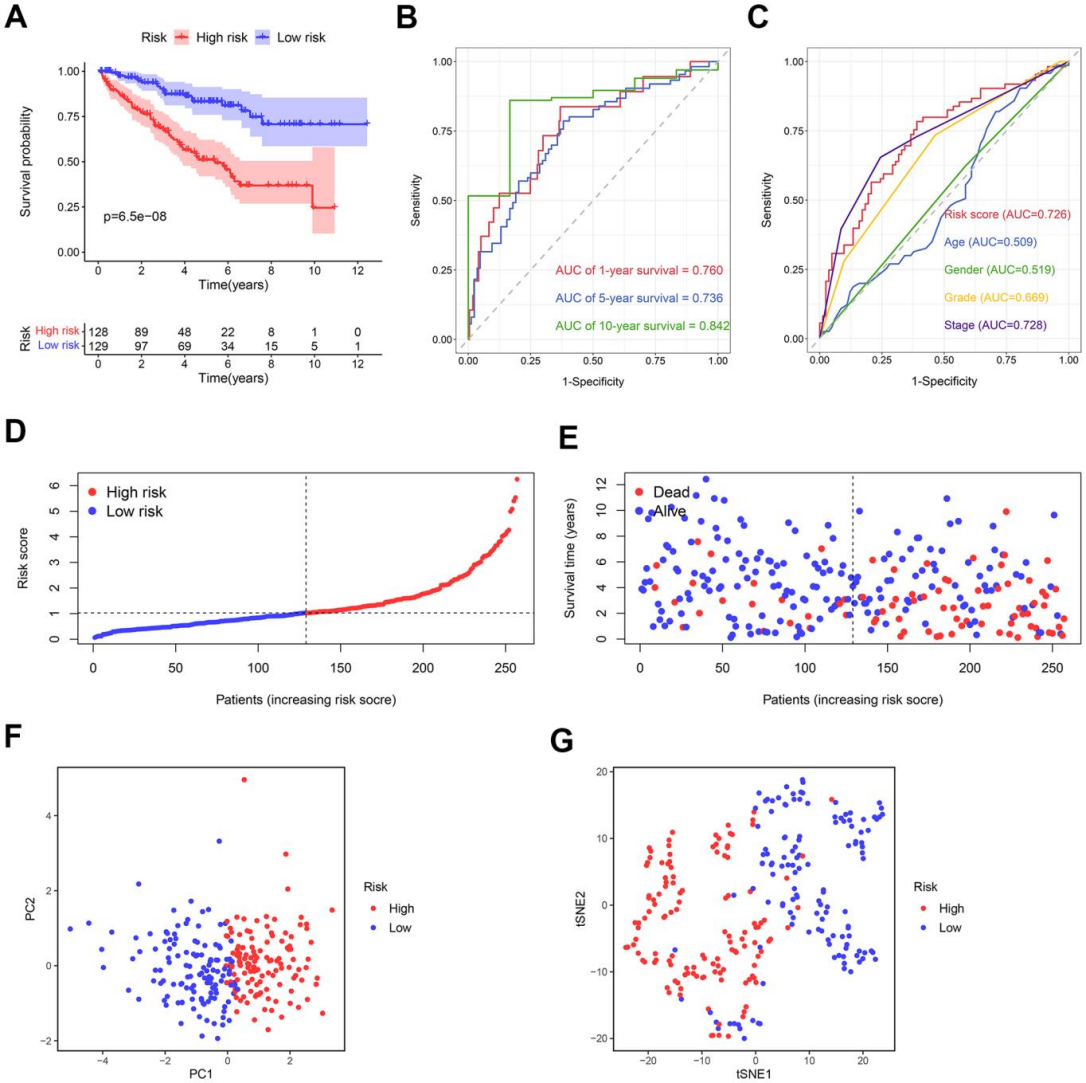
**The predictive ability of the prognostic risk model in the training cohort.** (**A**) The OS in the high- and low-risk groups. (**B**) ROC curves based on the prognostic risk model for predicting the 1-, 5-, and 10-year OS. (**C**) ROC curves based on the prognostic risk model and clinical parameters for predicting the 5-year OS. (**D**) Distribution of the risk score. (**E**) Correlation between the survival status and the risk score. (**F**) PCA for the high- and low-risk groups. (**G**) t-SNE analysis for the high- and low-risk groups.

For this purpose, each ccRCC sample was evaluated in terms of the risk score. The training cohort samples were categorized into the high- (n = 128) and low-risk (n = 129) groups using the median risk score = 4.575. Kaplan-Meier survival analysis revealed a significantly higher overall survival (OS) in the low-risk group compared to the high-risk one (Figure 7A). The predictive capacity of this prognostic risk model and clinical parameters were evaluated using the receiver operator characteristic (ROC) curves. The area under the curves (AUCs) of risk scores for 1-year, 5-year, and 10-year survival were 0.760, 0.736, and 0.842, respectively (Figure 7B). Risk score, age, gender, grade, and stage AUCs at 5 years were 0.726, 0.509, 0.519, 0.669, 0.728, respectively (Figure 7C). The survival risk plot showed a poorer prognosis in the high-risk group. In other words, prognosis deteriorated with an increased risk score (Figure 7D, 7E). Both the principal component analysis (PCA) and t-distributed stochastic neighbor embedding (t-SNE) test showed samples from different risk groups were distributed in different sections (Figure 7F, 7G).

### Evaluation of the prognostic signature in the testing cohort

**Figure 8:**
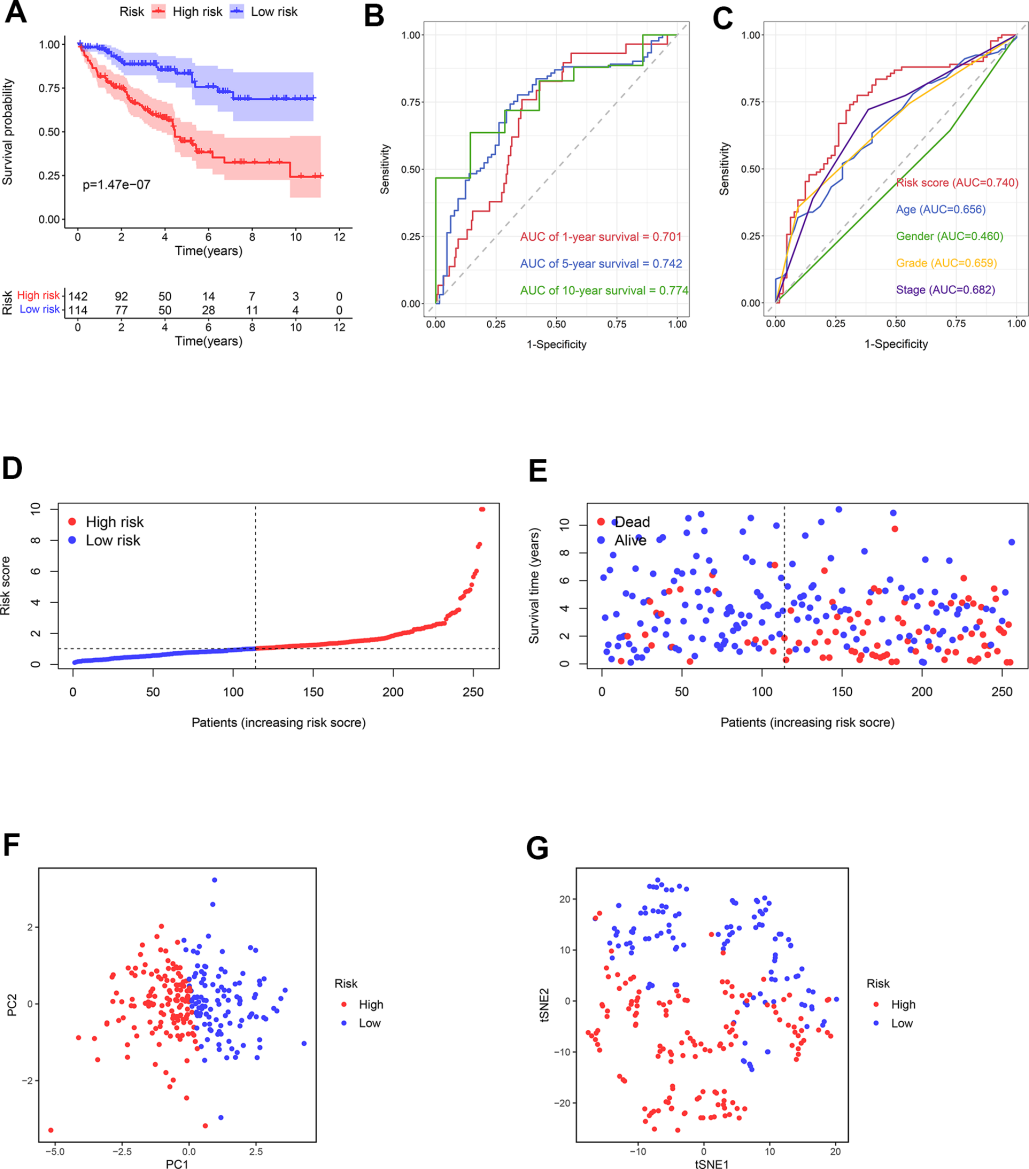
**The predictive ability of the prognostic risk model in the testing cohort.** (**A**) The OS in the high- and low-risk groups. (**B**) ROC curves based on the prognostic risk model for predicting the 1-, 5-, and 10-year OS. (**C**) ROC curves based on the prognostic risk model and clinical parameters for predicting the 5-year OS. (**D**) Distribution of the risk score. (**E**) Correlation between the survival status and the risk score. (**F**) PCA for the high- and low-risk groups. (**G**) t-SNE analysis for the high- and low-risk groups.

Using the median risk score of the training cohort, the ccRCC samples in the testing cohort could be categorized as the high- (n = 142) and low-risk (n = 114) groups. Consistent with the previous results, the prognosis in low-risk samples was better than that of the high-risk samples (Figure 8A). The AUCs of risk scores for 1-year, 5-year, and 10-year survival were 0.701, 0.742, and 0.774, respectively (Figure 8B). The predictive performance of the present risk score was more precise than clinical parameters and even other previous models [20–23] (Figure 8C, Supplementary Figure 1). The survival risk plot confirmed a more favorable prognosis in the low-risk group compared to the high-risk one (Figure 8D, 8E). The PCA and t-SNE analyses distributed the groups with distinct risk scores into two different categories (Figure 8F, 8G).

### Validation of independent prognostic factors

**Figure 9:**
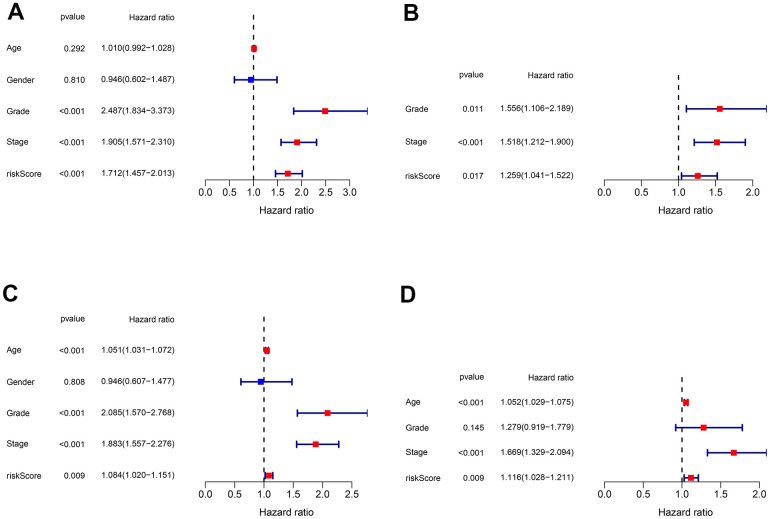
**Validation of the independent prognostic factors.** (**A**, **B**) Univariate and multivariate Cox regression analyses of the risk score and clinical parameters in the training cohort. (**C**, **D**) Univariate and multivariate Cox regression analyses of the risk score and clinical parameters in the testing cohort.

To discover if the risk score acts as an independent prognostic clinical parameter, the univariate and multivariate Cox regression analyses were performed. The results confirmed that the risk score and the stage as genuine independent prognostic OS predictors in both training cohort (Figure 9A, 9B) and testing cohort (Figure 9C, 9D).

### Identification of the genes related to the high-risk group

**Figure 10:**
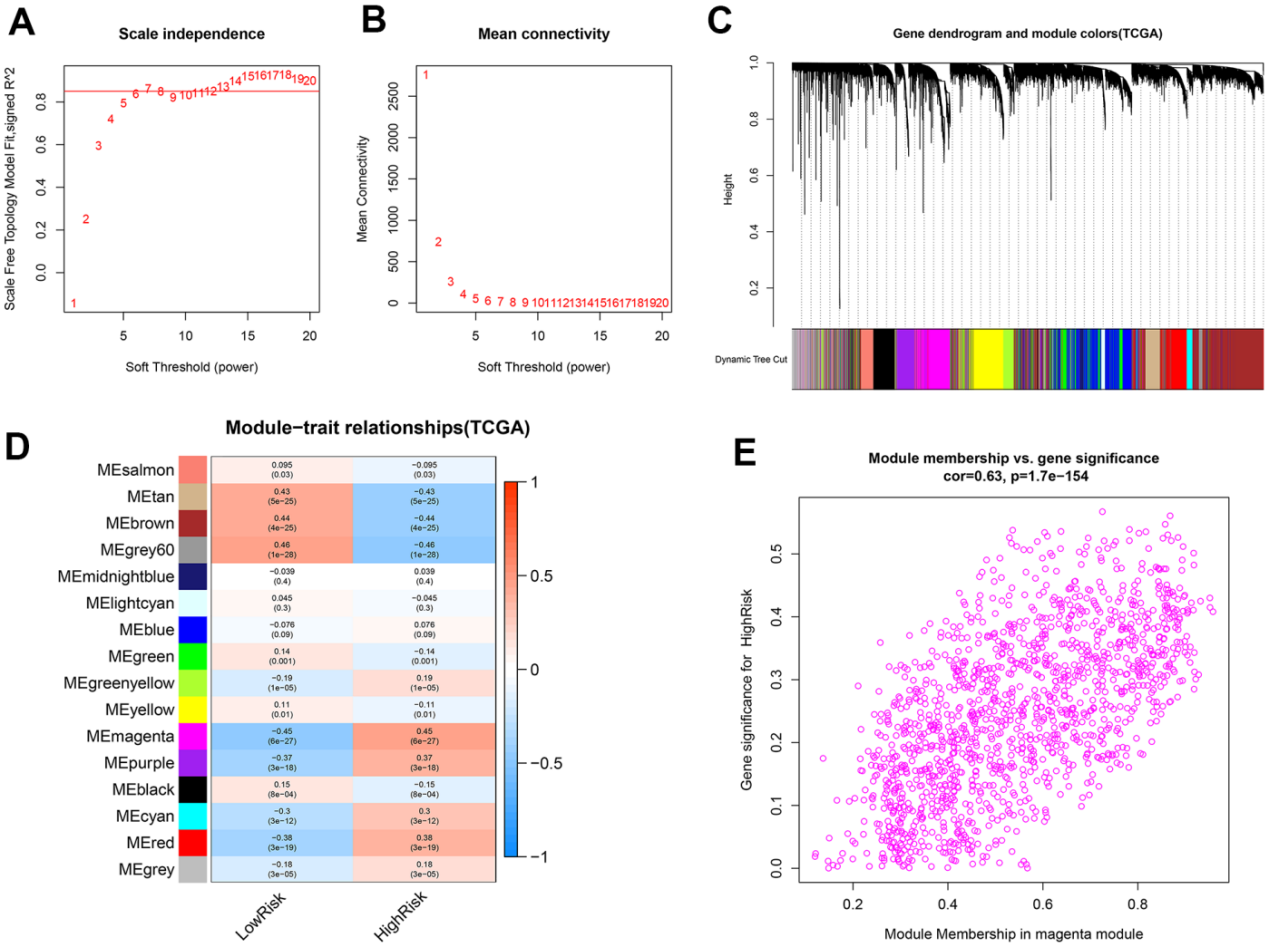
**Identification of co-expression modules related to the high-risk group by WGCNA.** (**A**, **B**) The scale-free fit index and the mean connectivity for various soft-thresholding powers (β) were optimized. (**C**) Cluster dendrogram of genes based on the 1-TOM. (**D**) Heatmap of the correlation between module eigengenes and sample types. (**E**) Scatter plot of module eigengenes for the high-risk group in the magenta module.

WGCNA was applied to identify the genes related to the high-risk group. For this purpose, a soft-threshold β = 7 (R^2^ = 0.86) was opted for developing a scale-free network (Figure 10A, 10B). Accordingly, 16 modules were identified to have a positive correlation with the high-risk group, among which the magenta module had the highest correlation value (Figure 10C, 10D). The correlation between MM and GS was 0.63 (Figure 10E). Finally, 1390 mRNAs in the magenta module were selected for functional enrichment analysis.

### Functional enrichment exploration of the high-risk group related genes

**Figure 11:**
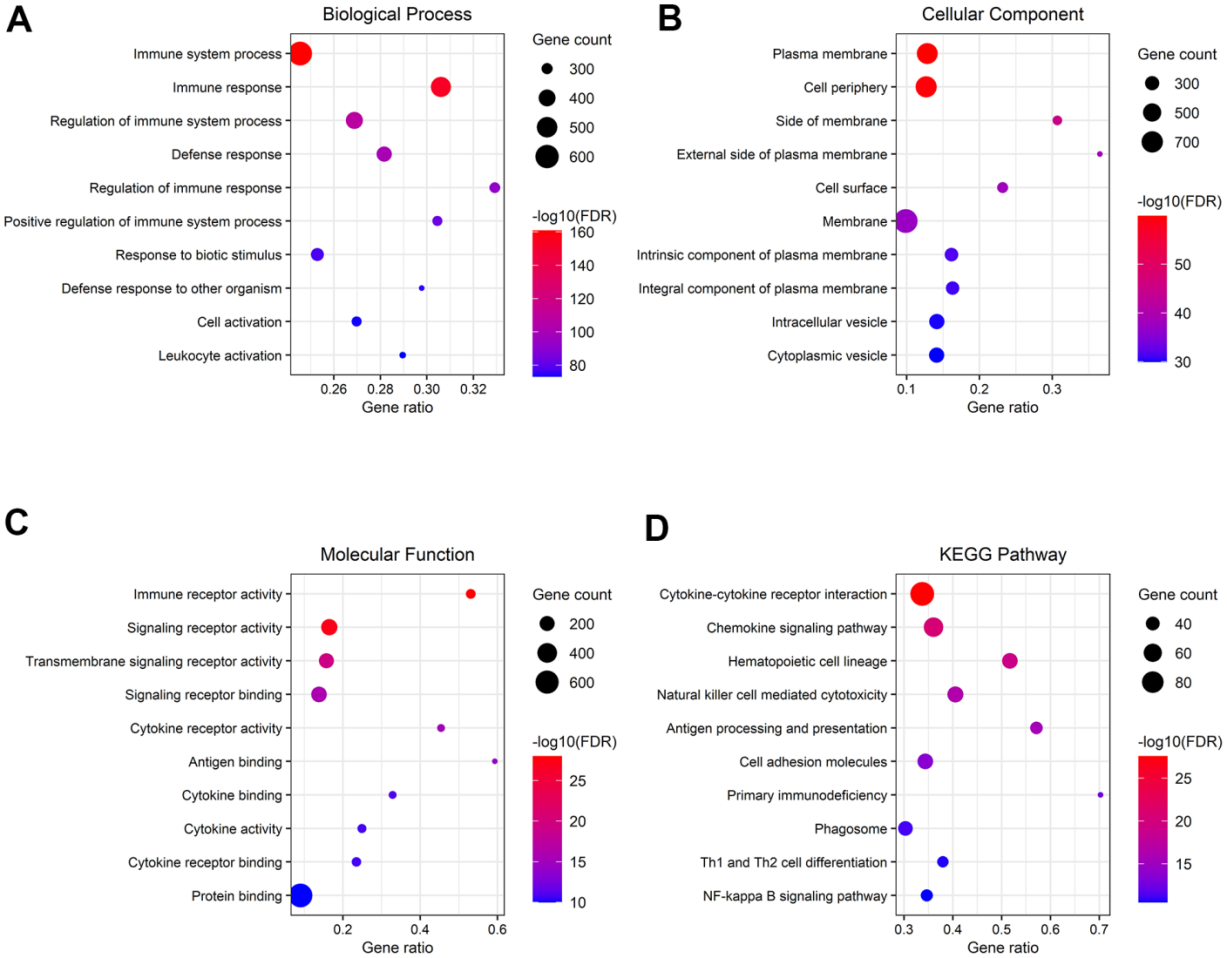
**Gene functional enrichment analysis of genes associated with the high-risk group.** (**A**–**C**) Overrepresented GO terms for BP, CC, and MF. (**D**) Top 10 enriched functional pathway terms based on KEGG analysis.

To disclose the functions and pathways involved in samples of the high-risk group, GO and KEGG pathway enrichment analyses were applied on genes that were previously indicated to have the highest association with the high risk of ccRCC. BP terms included “immune system process”, “immune response”, and “regulation of immune system process” (Figure 11A). The genes related to CC were enriched in “plasma membrane”, “cell periphery”, and “side of membrane” (Figure 11B). In MF, the genes were involved in “immune receptor activity”, “signaling receptor activity”, and “transmembrane signaling receptor activity” (Figure 11C). Pathway analysis with KEGG illustrated that genes in “cytokine-cytokine receptor interaction”, “chemokine signaling pathway”, and “natural killer cell-mediated cytotoxicity” were significantly enriched (Figure 11D).

### Identification of immune cell infiltration in ccRCC for different risk scores

**Figure 12:**
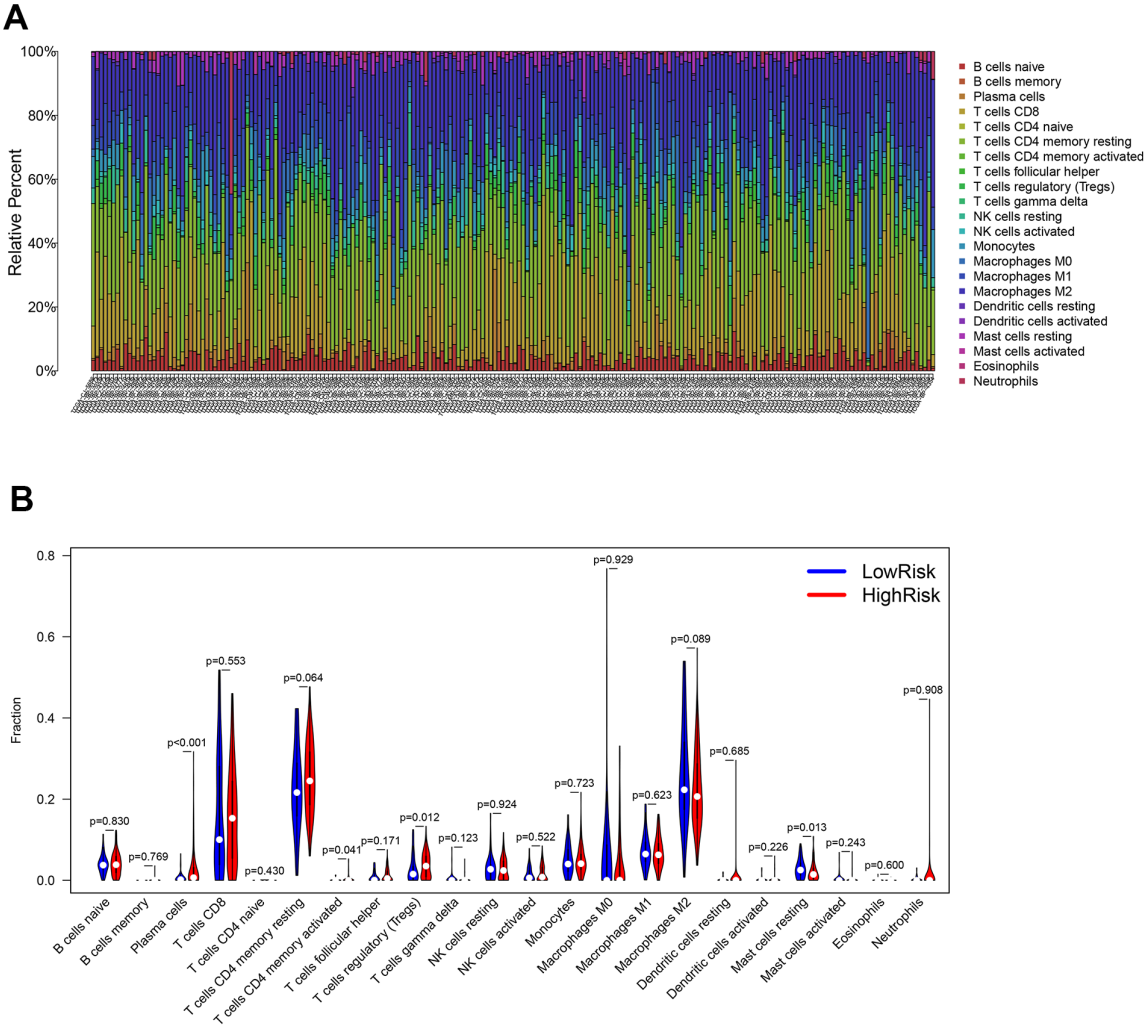
**Evaluation of the immune cell infiltration in ccRCC samples.** (**A**) The abundance of 22 immune cell types in ccRCC samples. (**B**) The proportion of different immune cell types between the high- and low-risk groups in ccRCC samples.

The association between the infiltration levels of immune cells and the risk score was investigated by measuring the abundance of 22 different types of immune cells in ccRCC samples using the CIBERSORT algorithm (Figure 12A). The differential distribution of immune cell types between the groups with high and low risks of ccRCC was also examined. The results indicated a significantly greater proportion of infiltration of plasma cells, CD4 memory-activated T cells, and regulatory T cells (Tregs) in the high-risk group compared to the low-risk group. In contrast, the infiltration of resting mast cells in the high-risk group was substantially lower than the same parameter in the low-risk group (Figure 12B).

### Identification of ICB-related gene expression for different risk scores

**Figure 13:**
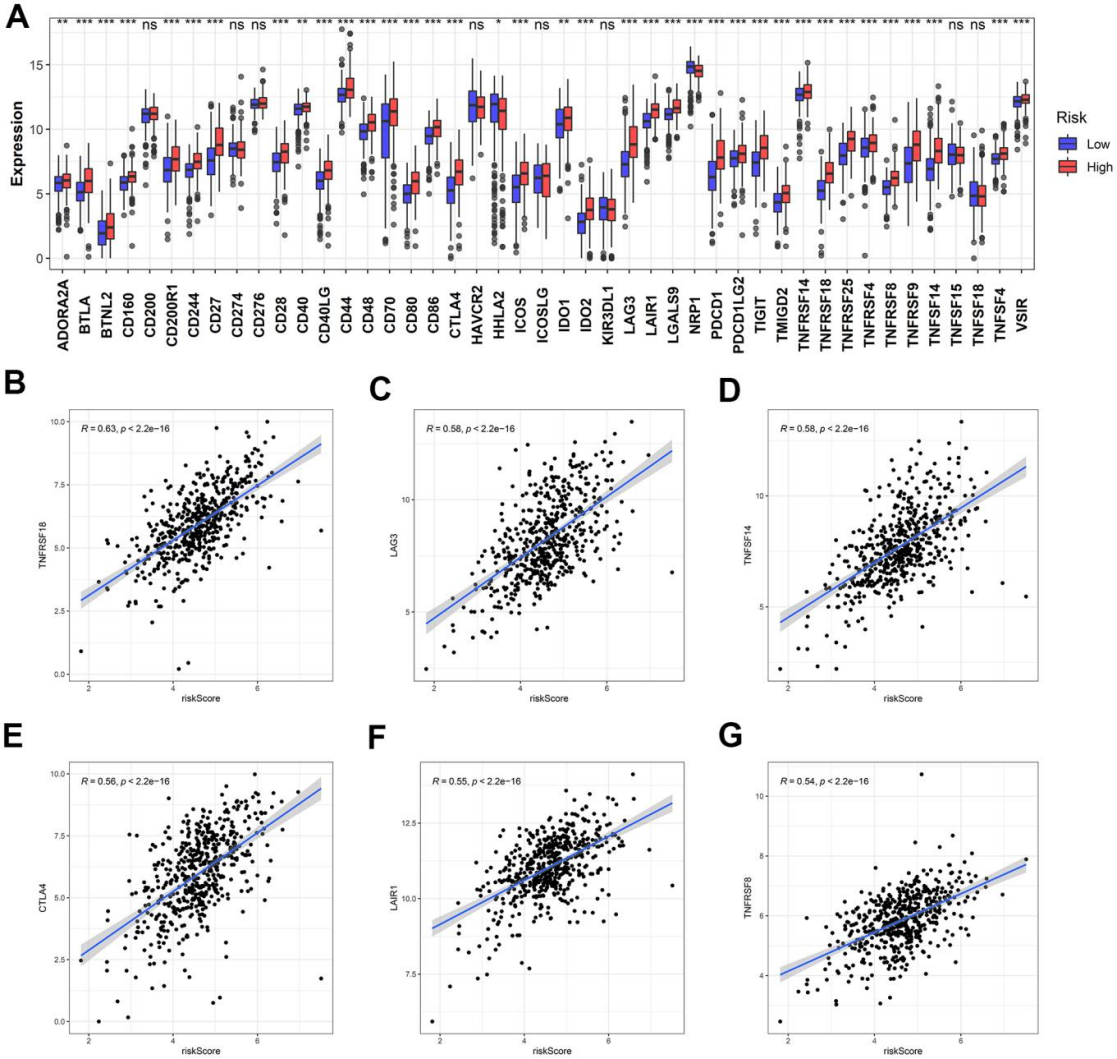
**Identification of the correlation between the risk score and ICB-related genes.** (**A**) The differential expression of ICB-related genes between the high- and low-risk groups. (**B**–**G**) The top six ICB-related genes with the most relevance to the risk score.

Since ICB-related genes are widely known for their involvement in the development of cancers, the expressions of 47 ICB-related genes were also analyzed in the ccRCC samples with different risk scores. The expressions of multiple ICB-related genes in the low-risk group were significantly lower than that in the high-risk group (Figure 13A). Moreover, the expressions of most ICB-related genes and risk scores were positively correlated (Supplementary Table 1). The top six genes highly correlated with the risk score are shown in Figure 13B–13G.

## DISCUSSION

The recently developed high-throughput sequencing technologies have shifted the research focus from protein-coding RNAs to ncRNAs, especially lncRNAs. These molecules are being rapidly identified and characterized by the accumulating evidence to be strongly associated with tumorigenesis and progression in diverse cancers [24]. In addition, as compared to protein-coding RNAs, the relationship between lncRNAs and cancer status is tighter, which implies that lncRNAs may serve as accurate diagnostic, therapeutic, and prognostic biomarkers of several malignancies [25]. For instance, lncRNA SARCC expression in ccRCC tissues decreases, and low SARCC expression is associated with a worse prognosis. Androgen receptor has been identified as a target gene of SARCC. Knocking down SARCC decreases the expression of miR-143-3p and stimulates multiple oncogenes by enhancing androgen receptor expression, which leads to greater cancer cell proliferation, migration, and invasion [26]. Similarly, lncRNA MRCCAT1 is overexpressed in ccRCC cell lines as well as the relevant tissues. MRCCAT1 enhances the binding of PRC2 at NPR3 promoter regions, leading to transcriptional repression of NPR3 and the activation of the p38-MAPK signaling pathway. Thus, the aggressive cancerous cell phenotypes are enhanced both *in vitro* and *in vivo* [27]. Although previous studies have expanded our knowledge on lncRNAs to a certain extent, the potential functions and molecular mechanisms underlying lncRNA actions remain unknown.

WGCNA package in R is widely used for identifying the co-expressed gene modules based on the correlation within gene expression profiles as well as the correlation between gene modules and clinical characteristics. Thereby, the genes with analogous expression tendencies and functions are clustered into the separate gene modules, and those gene modules which are most relevant to clinical traits are representative of the prominent regulators of the disease [28, 29]. The differential gene expression analysis and WGCNA are performed for genes associated with ccRCC, and the overlapping genes are considered to be pivotally involved in the development of ccRCC. To the best of our knowledge, the present report is the first study that employs the results of WGCNA to construct the ceRNA network in ccRCC. Thus, our results are reliable and convincing.

The role of immune system involvement in carcinogenesis is widely recognized. In immunocompetent individuals, the immune system takes responsibility for recognizing and exterminating the cancer cells [30]. However, emerging evidence suggests that evasion of immune destruction is a distinct feature of cancers [31]. Hence, immunotherapy as a promising therapeutic strategy has attracted extensive research attention. Several immunotherapeutic biomarkers have been identified, and the corresponding immunotherapeutic agents have received approval to be used in clinical trials or treatment, including cytotoxic T-lymphocyte associated protein 4 (CTLA4) inhibitor, programmed cell death protein 1 (PD-1)/ PD-1 ligand 1 (PD-L1) inhibitor, and chimeric antigen receptor (CAR) T cell. Nevertheless, such treatments are currently accessible only for a specific subset of patients. Thus, the identification of novel immunotherapeutic biomarkers is necessary [32]. Accumulating studies suggest that lncRNAs may function as effective immunotherapeutic targets. Huang et al. [33] reported that activation-induced cell death of T-lymphocytes assists cancer cell evasion from immune destruction. lncRNA NKILA overexpression sensitizes tumor-specific cytotoxic T-lymphocytes and type 1 helper T cells, resulting in activation-induced cell death and cancer immune evasion in breast and lung cancers. Liu et al. [34] reported that LINC00973 induces immune suppression by regulating the ceRNA network. Siglec-15, a novel immune suppressor, is promoted by LINC00973/miR-7109 axis and leads to immune evasion in ccRCC. Consequently, these results imply that lncRNAs in the ceRNA network may regulate immune-related genes and provide novel immunotherapeutic targets. In our study, we identified four promising lncRNAs including LINC00943, SRD5A3-AS1, LINC02345, and U62317.3. A recent study shows that LINC00943 is upregulated in gastric cancer. Knocking down LINC00943 enhances the expression of miR-101-3p, resulting in the suppression of cell proliferation and chemoresistance. However, SRD5A3-AS1, U62317.3, and LINC02345 have not yet been reported in cancers.

Immune cell infiltration is a pivotal component of TME, and dysregulation of immune cells has a powerful impact on clinical outcomes. The present results demonstrated that the immune cell infiltration is distinguished and distinctive in ccRCC samples belonging to different risk groups. In samples with high risk scores, plasma cells, CD4 memory-activated T cells, and Tregs were upregulated, while resting mast cells were downregulated. Several studies report high immune cell infiltration in ccRCC, especially that of the T cells [35, 36]. Upregulation of CD4 memory-activated T cells and Tregs showed to be negatively correlated with the OS [37]. Tregs are potent immunosuppressive cells that limit antitumor immunity and promote angiogenesis. Extensive Tregs infiltration is reported in multiple tumor types and is linked with poor clinical outcomes [38]. B cells can differentiate into plasma cells and produce antibodies, and are an important component of TME [39]. However, B cells recruited from TME promote RCC metastasis by activating the IL-1β/HIF-2α/Notch1 signaling axis [40]. The role of mast cells in ccRCC remains controversial. Some studies show that mast cell infiltration correlates with poor survival, since it has shown a positive correlation with the size, grade, and metastasis parameters of the tumor [41, 42]. Nevertheless, Xiong et al. [43] suggest that increased infiltration of mast cells represents high sensitivity to tyrosine kinase inhibitors treatment response and good survival. The controversy is reflective of the inherent complexity of TME in ccRCC, and more studies are required to elucidate and address the TME complexities in the future.

In conclusion, an immune signature-related ceRNA network was constructed, and a corresponding prognostic risk model was developed based on four lncRNAs with a significant prognostic value. To achieve this, the ccRCC samples have been categorized into the high- and low-risk groups according to the risk score. Further analyses indicated that the clinical outcome was significantly correlated with specific immune cell infiltration levels and ICB-related gene expression in ccRCC samples belonging to different risk groups. Therefore, we envisage that our study offers a reference for identifying novel prognostic biomarkers and benefits studies aimed at developing immunotherapeutic strategies.

## MATERIALS AND METHODS

### Data collection

TCGA is a cancer genomics consortia-developed database comprising more than 20,000 primary cancer and correspondingly matched normal tissues from patients, spanning across 33 different cancer types. The TCGA portal (https://portal.gdc.cancer.gov/) was used to retrieve the RNA sequences and clinical data of ccRCC samples. The downloaded lncRNA and mRNA sequencing data comprised 539 ccRCC samples and 72 normal tissue samples of the kidney. The miRNA sequencing data comprised 545 ccRCC and 71 normal tissue samples of the kidney. Since the required data were accessible from a public portal, no ethical approval or informed consent was needed.

### Differential gene expression analysis

The gene sequencing data retrieved from TCGA were analyzed by the “edgeR” package [44] in the R software. The differentially expressed lncRNAs, miRNAs, and mRNAs were identified using |log2FC| > 1 and FDR < 0.05 as the set threshold.

### Construction of weighted gene co-expression network

WGCNA [45] was utilized to develop the gene co-expression modules gleaned from the clinical features. First, the gene expression profiles were normalized using the “edgeR” package. Then, data reliability confirmation was performed by checking the sample quality using the goodSamplesGenes function in the WGCNA package. Next, a soft threshold power β was opted for constructing a standard scale-free network. Subsequently, the correlation of genes was calculated using a power function, and an adjacency matrix was thus established. Thereafter, the obtained adjacency matrix underwent remodification into a topological overlap matrix (TOM). Genes then underwent the hierarchical clustering analysis based on dissimilarity (1-TOM), and the dynamic tree cut algorithm was used to seek the modules. Eventually, we applied a height cut-off of 0.25 for the modular dendrogram to merge modules with the most similarities.

### Conversion of gene names

The Ensembl database (http://www.ensembl.org/index.html) was used to convert the gene names from Ensembl gene stable ID to gene symbol [46].

### Construction of the ceRNA network

Firstly, the overlapping lncRNAs, miRNAs, and mRNAs between those from differential gene expression analysis and WGCNA were selected. Secondly, the genes targeted by lncRNAs were predicted using LncBase v.2 (http://carolina.imis.athena-innovation.gr/diana_tools/web/index.php?r=lncbasev2%2Findex-predicted) [47] and matched with corresponding differentially expressed miRNAs. Thirdly, the miRNAs-targeted genes were predicted using three databases, including TargetScan (http://www.targetscan.org/vert_72/) [48], miRDB (http://mirdb.org/) [49], and miRTarBase (http://mirtarbase.cuhk.edu.cn/php/index.php) [50]. The common predicted genes by these databases were selected and matched with differentially expressed mRNAs. Finally, we constructed and visualized the ceRNA network using Cytoscape version 3.8.0.

### Gene functional enrichment analysis

Using the STRING database, GO and KEGG pathway analyses were conducted to determine the overrepresented biological functions of genes in the ceRNA network (https://www.string-db.org/). GO terms describe the biological functions in three aspects: MF, CC, and BP [51]. KEGG is a database for the identification of high-level functions of a biological system with great utilities [52].

### Construction and validation of the prognostic risk model

The lncRNAs-based survival analysis in the ceRNA network was conducted to indicate the independent prognostic factors in ccRCC. For this purpose, the ccRCC clinical data were acquired from TCGA. Then, samples with a survival time of < 30 days were excluded. Next, the ccRCC samples were randomly grouped into the training (n = 257) and testing cohorts (n = 256) using the “caret” package. Next, LASSO regression and Cox regression analyses were used for the training cohort lncRNAs and a prognostic risk model was developed. Then, the samples in training or testing cohort were divided into the high- and low-risk groups based on the median risk score of the training cohort. Subsequently, the Kaplan-Meier survival analysis was used to measure the survival differences between the high- and low-risk groups. The ROC curves were plotted using the “timeROC” [53] package to evaluate the risk model performance by calculating the corresponding AUCs. The PCA and t-SNE analyses were conducted for assessing the distribution pattern of each risk group using “stats” and “Rtsne” packages. Finally, the univariate and multivariate Cox regression analyses were conducted to evaluate if the risk score serves as an independent prognostic factor in comparison to other clinical parameters.

### Analysis of the infiltration levels of immune cells

The infiltration levels of different immune cells were estimated by the CIBERSORT algorithm [54]. The leukocyte gene expression matrix was employed to identify the 22 immune cell types and their infiltration proportions in ccRCC samples with different risk scores. 1000 permutation count and p < 0.05 were set as the threshold.

### Analysis of the ICB-related genes

The differential expression of 47 ICB-related genes between the high- and low-risk samples, and the correlation of ICB-related genes expression and the risk score were evaluated. The results were visualized using the “ggpubr” package. p-value < 0.05 was considered to be statistically significant.

## Supplementary Material

Supplementary Figure 1

Supplementary Table 1
