# Supplementary material for: Identification of a competing endogenous RNA network related to immune signature in clear cell renal cell carcinoma
Source: Aging (Albany NY). 2021 Dec 27;13(24):25980–6002. doi: 10.18632/aging.203784 (PMC8751601; doi:10.18632/aging.203784)
Supplement: Supplementary Figure 1 [file aging-13-203784-s001.pdf]

SUPPLEMENTARY FIGURE

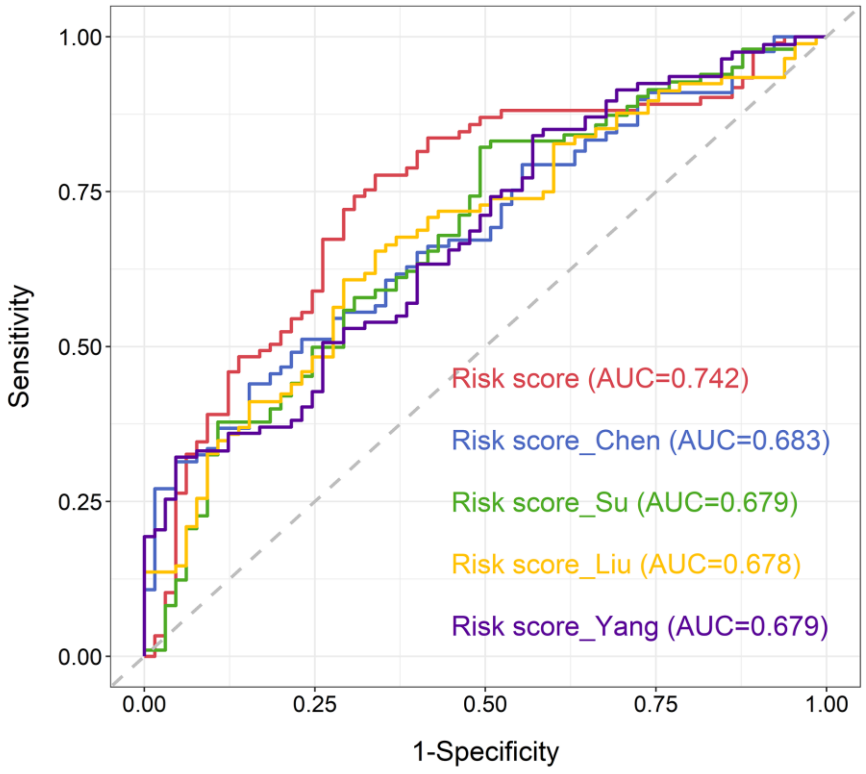

Supplementary Figure 1. ROC curves based on the present and previous prognostic risk models for predicting the 5-year OS in the testing cohort.
