# Supplementary material for: Identification of a competing endogenous RNA network related to immune signature in clear cell renal cell carcinoma
Source: Aging (Albany NY). 2021 Dec 27;13(24):25980–6002. doi: 10.18632/aging.203784 (PMC8751601; doi:10.18632/aging.203784)
Supplement: Supplementary Table 1 [file aging-13-203784-s002.pdf]

SUPPLEMENTARY TABLE

Supplementary Table 1. The correlation between the risk score and ICB-related genes.

Risk score	Genes	Correlation	Pvalue
Risk score	TNFRSF18	0.628998143	7.39E-58
Risk score	LAG3	0.582342029	6.63E-48
Risk score	TNFSF14	0.578299472	4.07E-47
Risk score	CTLA4	0.559690938	1.26E-43
Risk score	LAIR1	0.554739361	9.80E-43
Risk score	TNFRSF8	0.539604233	4.23E-40
Risk score	TIGIT	0.53570365	1.92E-39
Risk score	LGALS9	0.530655091	1.33E-38
Risk score	TNFRSF25	0.529224992	2.28E-38
Risk score	PDCD1	0.529215128	2.29E-38
Risk score	CD27	0.506205589	9.80E-35
Risk score	TNFRSF9	0.492734013	9.83E-33
Risk score	CD80	0.487758574	5.12E-32
Risk score	ICOS	0.462756108	1.38E-28
Risk score	CD244	0.461539911	1.99E-28
Risk score	IDO2	0.456579955	8.79E-28
Risk score	TMIGD2	0.433183535	7.03E-25
Risk score	CD40LG	0.4279925	2.89E-24
Risk score	CD86	0.420920703	1.91E-23
Risk score	CD48	0.408111515	5.21E-22
Risk score	TNFSF4	0.406643645	7.54E-22
Risk score	CD70	0.389525679	4.93E-20
Risk score	BTLA	0.387656784	7.67E-20
Risk score	TNFSF9	0.38474049	1.52E-19
Risk score	CD200R1	0.372174827	2.68E-18
Risk score	CD160	0.36218579	2.40E-17
Risk score	CD28	0.359554881	4.22E-17
Risk score	CD44	0.334013414	7.78E-15
Risk score	CD40	0.292971982	1.30E-11
Risk score	ADORA2A	0.254870413	4.75E-09
Risk score	PDCD1LG2	0.248463719	1.17E-08
Risk score	TNFRSF4	0.233256205	9.08E-08
Risk score	TNFRSF14	0.22041312	4.60E-07
Risk score	VSIR	0.220110972	4.78E-07
Risk score	BTNL2	0.183270027	2.96E-05
Risk score	IDO1	0.166154592	0.000156546
Risk score	CD276	0.15429199	0.000452959
Risk score	TNFSF18	0.069039149	0.118346459
Risk score	HAVCR2	0.062374443	0.158339645
Risk score	CD200	0.059886251	0.175640432
Risk score	HHLA2	0.036799909	0.405551096
Risk score	CD274	0.01448957	0.743366739

Risk score	KIR3DL1	0.003153622	0.943195674
Risk score	ICOSLG	-0.031925248	0.470595153
Risk score	NRP1	-0.044113629	0.318667462
Risk score	TNFSF15	-0.093830422	0.03361018
Risk score	VTCN1	-0.205208172	2.78E-06
